# Improving diagnostic sensitivity of combined dermoscopy and reflectance confocal microscopy imaging through double reader concordance evaluation in telemedicine settings: A retrospective study of 1000 equivocal cases

**DOI:** 10.1371/journal.pone.0187748

**Published:** 2017-11-09

**Authors:** A. M. Witkowski, J. Łudzik, F. Arginelli, S. Bassoli, E. Benati, A. Casari, N. De Carvalho, B. De Pace, F. Farnetani, A. Losi, M. Manfredini, C. Reggiani, J. Malvehy, G. Pellacani

**Affiliations:** 1 Department of Dermatology, University of Modena and Reggio Emilia, Modena, Italy; 2 Department of Biostatistics and Telemedicine, Jagiellonian University Medical College, Krakow, Poland; 3 Dermatology Department, Melanoma Unit, Barcelona, Spain; University of Queensland Diamantina Institute, AUSTRALIA

## Abstract

**Background:**

Reflectance confocal microscopy (RCM) is an imaging device that permits non-invasive visualization of cellular morphology and has been shown to improve diagnostic accuracy of dermoscopically equivocal cutaneous lesions. The application of double reader concordance evaluation of dermoscopy-RCM image sets in retrospective settings and its potential application to telemedicine evaluation has not been tested in a large study population.

**Objective:**

To improve diagnostic sensitivity of RCM image diagnosis using a double reader concordance evaluation approach; to reduce mismanagement of equivocal cutaneous lesions in retrospective consultation and telemedicine settings.

**Methods:**

1000 combined dermoscopy-RCM image sets were evaluated in blind by 10 readers with advanced training and internship in dermoscopy and RCM evaluation. We compared sensitivity and specificity of single reader evaluation versus double reader concordance evaluation as well as the effect of diagnostic confidence on lesion management in a retrospective setting.

**Results:**

Single reader evaluation resulted in an overall sensitivity of 95.2% and specificity of 76.3%, with misdiagnosis of 8 melanomas, 4 basal cell carcinomas and 2 squamous cell carcinomas. Combined double reader evaluation resulted in an overall sensitivity of 98.3% and specificity of 65.5%, with misdiagnosis of 1 *in-situ* melanoma and 2 basal cell carcinomas.

**Conclusion:**

Evaluation of dermoscopy-RCM image sets of cutaneous lesions by single reader evaluation in retrospective settings is limited by sensitivity levels that may result in potential mismanagement of malignant lesions. Double reader blind concordance evaluation may improve the sensitivity of diagnosis and management safety. The use of a second check can be implemented in telemedicine settings where expert consultation and second opinions may be required.

## Introduction

Cutaneous tumor diagnosis can be difficult due to the diverse clinical and dermoscopic presentation of cutaneous lesions. In order to correctly identify an early melanoma (MM) the use of dermoscopy has been shown to significantly increase the sensitivity and specificity of diagnosis when compared with traditional naked-eye examination [1,2]. In equivocal cases benign lesions may be excised when further cytological information is required to rule out malignancy. The efficacy of benign tumor differentiation from MM can be measured by the number needed to excise ratio (NNE), for which dermoscopy ranges between 8.7 to 29.4, according to the level of expertise and clinical setting [3,4].

In the past decade reflectance confocal microscopy (RCM) use in clinical practice has been shown to further improve early MM diagnosis and help to reduce the number of unnecessary excisions in different settings, as summarized in Table 1 [5–22] and confirmed by recent reviews and meta-analysis [23,24].

**Table 1:** Previous studies evaluating the accuracy of reflectance confocal microscopy.

ref. n.	First Author, year	Journal	Country	Setting	Total lesion number	Malignancies	Sensitivity	Specificity	Study population	Diagnostic reference
5	Łudzik, 2016	PLoS One	Poland, Italy	Retrospective	316	12 MMs, 138 BCCs, 20 SCCs	94% (Double reading 98%)	54% (Double reading 42%)	Pink lesions	Histopathology + ≥1 year follow-up
6	Borsari, 2016	JAMA Dermatol	Italy	**Prospective Interventional**	1279	246 MMs, 61 BCCs, 16 SCCs	97%	62%	Dermoscopically equivocal, any type	Histopathology + ≥1 year follow-up
7	Guitera, 2016	Br J Dermatol	Australia, Italy	Retrospective	191	45 MMs, 48 BCCs, 10 SCCs	84%	47%	Pink lesions	Histopathology
8	Stanganelli, 2016	J Eu Acad Dermatol Venereol	Spain	Retrospective	70	12 MMs	92%	46%	Melanocytic lesions changing at sequential digital dermoscopy	Histopathology
9	Lovatto, 2016	Br J Dermatol	Italy	Retrospective	64	13 MMs	100%	69%	Melanocytic lesions changing at sequential digital dermoscopy	Histopathology
10	Farnetani, 2015	JAMA Dermatol	International	Retrospective	100	20 MMs, 15 BCCs	89% (Majority 100%)	79% (Majority 80%)	Selected lesions evaluated by 9 evaluators	Histopathology
11	Pellacani, 2015	Br J Dermatol	Italy	**Prospective Interventional**	493	29 MMs, 39 BCCs	100%	47%	Dermoscopically equivocal lesions, any type	Histopathology + ≥1 year follow-up
12	Alarcon, 2015	Br J Dermatol	Spain	**Prospective Interventional**	343	92 MMs	100%	31%	Dermoscopically equivocal melanocytic lesions	Histopathology + ≥1 year follow-up
13	Ferrari, 2014	J Eu Acad Dermatol Venereol	Italy	Retrospective	322	70 MMs	96%	70%	Dermoscopically equivocal melanocytic lesions	Histopathology
14	Rao, 2013	J American Acad Dermatol	USA, Italy	Retrospective	334	9 MMs, 27 BCCs, 43 SCCs	98%	44%	Dermoscopically equivocal melanocytic and non-melanocytic lesions	Histopathology
15	Longo, 2013	Br J Dermatol	Italy	Retrospective	140	32 MMs, 34 NMSCs	96%	94%	Dermoscopically equivocal nodular lesions	Histopathology
16	Guitera, 2012	J Invest Dermatol	Italy, Australia	Retrospective	710	216 MMs, 119 BCCs	88%	70%	Clinically/dermoscopically equivocal melanocytic and non-melanocytic lesions	Histopathology
17	Guitera, 2010	J Invest Dermatol	Italy, Spain, Australia, USA	Retrospective	219	81 LMs	85%	76%	Facial lesions	Histopathology
18	Guitera, 2009	J Invest Dermatol	Italy, Australia	Retrospective	326	123 MMs	91%	68%	Dermoscopically equivocal melanocytic lesions	Histopathology
19	Segura, 2009	J American Acad Dermatol	Spain	Retrospective	154	36 MMs, 27 BCCs	100%	57%	Dermoscopically equivocal melanocytic and non- melanocytic lesions	Histopathology
20	Langley, 2007	Dermatology	Canada	**Prospective Non-Interventional**	125	37 MMs	97%	83%	Clinically equivocal melanocytic lesion	Histopathology
21	Pellacani, 2007	J Invest Dermatol	Italy	Retrospective	351	136 MMs	92%	69%	Dermoscopically equivocal melanocytic lesions	Histopathology
22	Pellacani, 2005	J American Acad Dermatol	Italy	Retrospective	102	37 MMs	97%	72%	Dermoscopically equivocal melanocytic lesions	Histopathology

MM–melanoma, BCC–basal cell carcinoma, SCC–squamous cell carcinoma

Recently, the reliability of teleconsultation with dermoscopy images only [25,26] or combined dermoscopy-RCM images [5,10,14] has been tested which showed the capability of an accurate diagnosis but risk of mismanagement of potentially dangerous lesions, with diagnostic accuracy depending greatly on the level of expertise. Since RCM is a newly adopted technology its limitation to proper and safe diffusion may be due to the limited number of expert users in the field and lack of dedicated training programs to properly support the necessary knowledge acquisition and experience needed for safe and effective implementation in clinical practice. With the potential for an increase in requests for distant expert consultation of RCM images there is a need to provide proper, accurate and safe management of lesions sent for second expert consultation and in addition provide an available and effective training program for new users. Our goal in this study was to test the diagnostic sensitivity of readers who followed a dedicated dermoscopy-RCM training program and evaluate the difference between single reading versus double reading concordance evaluation of dermoscopy-RCM image sets. Additionally, we intended to determine if it is possible to reduce mismanagement of equivocal cutaneous lesions in retrospective consultation and telemedicine settings.

## Materials and methods

### Patient population

This was an Ethical Committee approved retrospective study (protocol 71.14; November 11, 2015) within the European Project DIAGNOPTICS (grant n. 621066) based on 1000 consecutive cases that were evaluated with dermoscopy and RCM imaging for diagnostic decision in order to rule out a diagnosis of MM during the period of January 2010 to August 2011 at the Dermatology Department at the University of Modena and Reggio Emilia (UNIMORE). The inclusion criteria were: (i) lesion excised during the first visit or follow-up sequential digital dermoscopy (i.e. videodermoscopy; mole-mapping) control visit with corresponding histology report; (ii) lesion not excised but with at least a 1 year stable digital dermoscopy and RCM imaging follow-up with no significant structural changes in dermoscopy and absence of increased atypia in RCM–considered to be benign and comparable to the histopathology gold standard; (iii) availability of digital dermoscopy images; (iv) availability of a complete standard set of RCM images. The first 1000 cases fitting the inclusion criteria were selected for this study in order to obtain an even number of cases to be evaluated by the participating readers. All patient cases were organized and renamed (0001–1000) by AW into separate folders without the patient name, number or identifying information so that the participating readers were blinded from the actual diagnosis and treatment course listed in the department database.

### Reader training

Ten readers that included eight dermatology residents and two newly trained dermatology specialists participated in a tutorial training program for a six month duration with a minimum of 2000 dermoscopy-RCM case reviews and at least three months of clinical exposure to bedside dermoscopy-RCM clinical decision making at the UNIMORE skin cancer unit.

### Imaging protocol and evaluation

Dermoscopy images were obtained with a DermLite FOTO System (DermLite Photo; 3Gen, San Juan Capistrano, CA, USA). RCM images were obtained with a reflectance confocal microscope (Vivascope 1500; MAVIG GmbH, Munich, Germany). A minimum of three mosaics (VivaCube; Caliber I.D., Inc., Rochester, NY, USA), images of 0.5 mm x 0.5 mm acquired and stitched into composite images, were obtained at different depths, corresponding to the stratum granulosum/spinosum, the dermal-epidermal junction (DEJ) and the papillary dermis [22]. The 1000 consecutive cases selected for evaluation in this study included the following malignancies: MM, basal cell carcinoma (BCC) and squamous cell carcinoma (SCC) and benign lesions (including naevi, solar lentigos (SL), seborrheic keratosis (SK), lichen planus-like keratosis (LPLK) and actinic keratosis (AK)), or other benign lesions that were not used during the training period of the 10 readers. The image sets were made available for evaluation during a time period of 3 months in which each reader received a total of 200 randomized cases of dermoscopy-RCM image sets. Cases were randomized so that each case was evaluated in blind by two different readers. None of the readers obtained an identical case list and each case was only evaluated twice. Each reader was asked to provide their management decision, degree of confidence and suspected diagnosis based only on the provided image sets into a Microsoft excel file. Management recommendation (hypothetical) was grouped into two categories: (i) excision or (ii) no-excision. Management decision confidence level was graded: (i) low or (ii) high. In order to test concordance of double reading, data from the excel files (Readers 1 to 10) were matched by computer and automatically classified for management with excision when (1) management decision was concordant for excision or when (2) management decision was discordant (i.e. one reader was recommending excision and the other no-excision).

### Statistical analysis

Statistical analyses were performed using IBM SPSS Statistics Professional software (release 20.0.0, 2011; SPSS Inc., Chicago, IL, U.S.A.). Mean and standard deviation were calculated for Breslow’s thickness. Diagnostic values of sensitivity and specificity of individual readers were calculated for malignant versus benign lesions. Receiver Operating Characteristic (ROC) was calculated using binary diagnosis values (0: all benign lesion types, 1: all malignant lesion types) as the state variable and combination of “confidence” and “management” obtained by the 2 readers, corresponding to the following scores: -3 high confidence for no-excision of both readers; -2 no-excision suggested by both reader, one with high confidence and the other with low confidence; -1 low confidence for no-excision of both readers; 0 disagreement (one reader suggesting excision, while the other no-excision); +1 low confidence for excision of both readers; +2 excision suggested by both reader, one with high confidence and the other with low confidence; +3 high confidence for excision of both readers.

## Results

Based on histologic diagnosis there were 176 malignant cases that included MM (83 cases, mean Breslow’s thickness: 0.537 mm; standard deviation: 0.693 mm), BCC (87 cases) and SCC (6 cases). The remaining 824 cases included 749 naevi, of which 16 were spitz naevi, SL/SK/LPLK/AK (58 cases) and other benign lesions that included dermatofibromas, angiokeratomas, angiomas and other benign lesions classified as other (17 cases). The evaluation of 200 cases by each of the ten confocal readers was compared with the actual diagnosis resulting in 2000 total evaluations (each case evaluated by 2 different readers). Overall reader diagnostic performance is reported in (Table 2) (S1 Table).

**Table 2:** Individual management performance of single reader evaluations.

Reader number	Overall Sensitivity	Overall Specificity	MM sensitivity	BCC sensitivity	SCC sensitivity	Naevi (no spitz) specificity	Spitz naevi specificity	SK/SK/ LPLK/AK/Other specificity
**R1**	23/25 (92.0%)	139/175 (79.4%)	13/14 (92.9%)	10/11 (90.9%)	N/A	130/163 (79.8%)	0/2 (0%)	7/8 (87.5%)
**R2**	31/33 (93.9%)	125/167 (74.9%)	15/17 (88.2%)	16/16 (100%)	N/A	112/146 (76.7%)	0/4 (0%)	13/16 (81.3%)
**R3**	35/35 (100%)	132/165 (80.0%)	15/15 (100%)	18/18 (100%)	2/2 (100%)	120/147 (81.6%)	0/3 (0%)	9/10 (90.0%)
**R4**	41/42 (97.6%)	132/158 (83.5%)	14/14 (100%)	24/25 (96%)	3/3 (100%)	116/138 (84.1%)	0/2 (0%)	9/10 (90.0%)
**R5**	40/40 (100%)	118/160 (73.8%)	22/22 (100%)	17/17 (100%)	1/1 (100%)	104/140 (74.3%)	0/5 (0%)	12/13 (92.3%)
**R6**	34/37 (91.9%)	128/163 (78.5%)	13/15 (86.7%)	20/21 (95.2%)	1/1 (100%)	113/143 (79.0%)	0/2 (0%)	12/15 (80.0%)
**R7**	31/31 (100%)	114/169 (67.5%)	17/17 (100%)	13/13 (100%)	1/1 (100%)	109/157 (69.4%)	0/2 (0%)	4/8 (50.0%)
**R8**	36/37 (97.3%)	109/163 (66.9%)	20/20 (100%)	15/16 (93.8%)	1/1 (100%)	99/144 (68.8%)	0/5 (0%)	7/10 (70.0%)
**R9**	36/42 (85.7%)	131/158 (82.9%)	19/21 (90.5%)	17/19 (89.5%)	0/2 (0%)	115/135 (85.2%)	2/5 (40.0%)	11/15 (73.3%)
**R10**	28/30 (93.3%)	129/170 (75.9%)	9/11 (81.8%)	18/18 (100%)	1/1 (100%)	116/153 (75.8%)	0/2 (0%)	9/11 (81.8%)
**Single** **Overall**	**335/352 (95.2%)**	**1257/1648 (76.3%)**	**157/166 (94.6%)**	**168/174 (96.5%)**	**10/12 (83.3%)**	**1134/1466 (77.4%)**	**2/32 (6.2%)**	**121/150 (80.6%)**
**Double** **Overall**	**173/176 (98.3%)**	**540/824 (65.5%)**	**82/83 (98.8%)**	**85/87 (97.7%)**	**6/6 (100%)**	**486/733 (66.3%)**	**0/16** **(0%)**	**54/75 (72%)**

MM–melanoma, BCC–basal cell carcinoma, SCC–squamous cell carcinoma, SK–seborrheic keratosis, SL–solar lentigo, LPLK–lichen planus like keratosis, AK–actinic keratosis, R–reader, N/A–not applicable.

After randomization each reader received an average of 35 malignancies in their case series (ranging from 25 to 42 malignancies) including an average of 16.4 MMs (ranging between 14 to 22 MMs per reader). The overall average sensitivity for single reader evaluation was 95.2% (ranging between 86 to 100%) and specificity was 76.3% (ranging between 67 to 84%). Excision was recommended with high confidence in 142/166 (85.5%) evaluations of 83 MMs, 83/87 (95.4%) BCCs, 4/6 (66.7%) SCCs and 81/749 (10.8%) of benign lesions. Seventeen misdiagnoses of a malignancy were given out of 352 evaluations of malignant lesions, including 9 times for MM diagnosis (one of which was recommended twice for no-excision), 6 times for BCC diagnosis (two of which were recommended twice for no-excision) and twice for SCC diagnosis. In the group of hypothetically mismanaged malignancies readers reported high confidence in 2 out of 6 misdiagnosed invasive MMs, 2 out of 3 misdiagnosed *in-situ* MMs, 3 out of 6 misdiagnosed BCCs and 1 out of 2 misdiagnosed SCCs.

**Fig 1:**
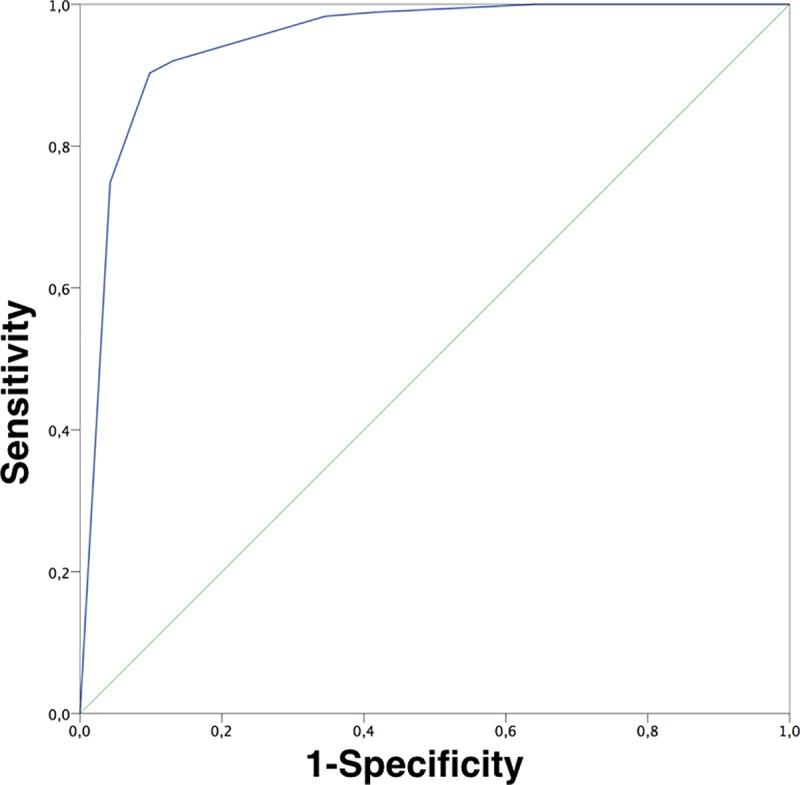
ROC curve for double reading.

Considering double reading the overall sensitivity was 98.3% and specificity was 65.5% for a cut-off threshold for excision when at least 1 reader suggested excision, regardless of their confidence level (Table 3). Only 1/83 (1.2%) MMs, corresponding to an *in-situ* MM, was mismanaged with concordant management decisions for no-excision by both readers. For BCCs 2/87 (2.3%) were mismanaged with concordant decisions of no-excision by both readers. For SCC excision was recommended in all cases. When two readers concordantly chose to manage a case with no-excision with both readers reporting high confidence no malignant lesions were mismanaged (35.4%, corresponding to 292 of 824 benign lesions). In all 3 mismanaged malignancies (1 *in-situ* MM and 2 BCCs) at least one reader reported a decision with low confidence. A discordant report (one reader suggesting excision and the other no-excision) was given for 12 malignancies (8 MMs, 2 BCCs and 2 SCCs) and 176 out of 824 benign lesions. Concordant agreement to excise resulted in correct management of 161/176 (91.4%) of malignancies and an over diagnosis of 108/824 (13.1%) benign lesions. ROC area under the curve for double reading diagnostic performance was 0.948 (p < 0.001) (Fig 1).

**Table 3:** Overall management performance of double reader evaluation.

	Double NEGATIVE (2 x HC) (-3)	Double NEGATIVE (1 x HC) (-2)	Double NEGATIVE (0 x HC) (-1)	1 POS & 1 NEG (0)	Double POSITIVE (0 x HC) (+1)	Double POSITIVE (1 x HC) (+2)	Double POSITIVE (2 x HC) (+3)	Total
**MM**	0	1	0	8	3	15	56	**83**
**BCC**	0	1	1	2	0	10	73	**87**
**SCC**	0	0	0	2	0	1	3	**6**
**Naevi (no spitz)**	255	178	53	161	23	40	23	**733**
**Spitz naevi**	0	0	0	2	3	2	9	**16**
**SK/SL/** **LPLK/AK**	28	11	2	11	1	3	2	**58**
**OTHER**	9	2	2	2	0	1	1	**17**

MM–melanoma, BCC–basal cell carcinoma, SCC–squamous cell carcinoma, SK–seborrheic keratosis, SL–solar lentigo, LPLK–lichen planus like keratosis, AK–actinic keratosis, HC–high confidence.

Individual RCM features were evaluated (based on 2000 evaluations) for their frequencies in the following categories: presence of pagetoid cells, architectural disarray with melanocytic features, BCC tumor islands and/or cords and marked dyskeratosis of the epidermis (Table 4). Pagetoid cells (atypical melanocytes at the level of the epidermis) were reported in 84.3% of MMs, 0.6% of BCCs and none of the SCCs. Pagetoid cells were also reported in benign lesions: 20.3% of naevi (excluding spitz naevi), 81.2% of spitz naevi, 8.8% of SL/SK/LPLK/AK and 2.7% of other benign lesions (p < 0.001). Architectural disarray with melanocytic features at the level of the dermal-epidermal junction was reported in 80.1% of MMs and was completely absent in both BCC and SCC. Frequencies of architectural disarray in benign lesions were the following: 16.6% of naevi (excluding spitz naevi), 43.8% of spitz naevi, 5.3% SL/SK/LPLK/AK and absent in other benign lesions (p < 0.001). For the evaluation of BCC tumor islands and/or cords their presence was reported in 89.1% of BCCs, 2.4% of MMs and 16.7% of SCCs. In all other benign lesions BCC tumor islands and/or cords were reported in less than 1% of cases (p < 0.001). Finally, marked dyskeratosis (greater than one third) of the epidermis was evaluated with the highest frequency in SCCs 58.3% and less than 10% in all other malignant and benign lesions (p < 0.001).

**Table 4:** Reported frequencies of specific RCM features (based on 2000 evaluations).

	MM	BCC	SCC	Naevi (no spitz)	Spitz naevi	SK/SK/ LPLK/AK	Other benign
**Pagetoid cells**	140/166 (84.3%)	1/174 (0.6%)	0/12 (0.0%)	297/1466 (20.3%)	26/32 (81.2%)	10/114 (8.8%)	1/36 (2.7%)
**Architectural** **Disarray with melanocytic features**	133/166 (80.1%)	0/174 (0%)	0/12 (0%)	243/1466 (16.6%)	14/32 (43.8%)	6/114 (5.3%)	0/36 (0%)
**BCC tumor islands/cords**	4/166 (2.4%)	155/174 (89.1%)	2/12 (16.7%)	7/1466 (0.48%)	1/32 (3.1%)	2/114 (1.8%)	1/36 (2.7%)
**Marked dyskeratosis** **(epidermis)**	13/166 (7.8%)	17/174 (9.8%)	7/12 (58.3%)	8/1466 (0.57%)	1/32 (3.1%)	11/114 (9.6%)	1/36 (2.7%)

MM–melanoma, BCC–basal cell carcinoma, SCC–squamous cell carcinoma, SK–seborrheic keratosis, SL–solar lentigo, LPLK–lichen planus like keratosis, AK–actinic keratosis.

## Discussion

The purpose of our study was to compare single reading versus double reading concordance evaluation of dermoscopy-RCM image sets. In the analysis we compared evaluations made by 10 readers to either histopathology diagnosis (excised cases) or what we considered benign lesions, those that were stable after one-year sequential digital dermoscopy in both dermoscopy and RCM imaging. We tested the diagnostic sensitivity and safety of evaluations in a retrospective and telemedicine setting, resulting in acceptable general diagnostic accuracy for both methods.

Unlike previous studies that tested the diagnostic accuracy of RCM based on single reader evaluations in non-telemedicine settings where one evaluation made on-site resulted in one simultaneous final management decision, we tested the possibility to implement RCM towards equivocal lesions that in real-world settings may be sent for a second opinion. By adding a second RCM reader and testing whether reader concordance and/or confidence has an impact on potential improvement of sensitivity we aimed to reduce the limitations of single RCM reader evaluation in both dermoscopic equivocal and confocal equivocal cases.

Previous RCM studies tested performance of single evaluators and reported variable sensitivities and specificities, mainly related to different study settings, study populations and evaluator expertise, but all studies demonstrated a good diagnostic performance in settings of difficult to diagnose lesions [5–22]. Previous studies include two main types: non-interventional and interventional studies. Non-interventional studies tested diagnostic accuracy in multiple study populations that in total included 3524 lesions, including 879 MMs. These studies mostly evaluated diagnostic performance of RCM alone and differential diagnosis was predominantly based on dermoscopically equivocal lesions [5,7–10,13–22]. Reported diagnostic sensitivity was high in all cases (ranging between 84 to 100%) and specificity variable, lower in more difficult study populations such as those containing pink lesions [5,7] and equivocal lesions that presented with architectural changes during sequential digital dermoscopic follow-up [8,9], demonstrating the usefulness and reliability of this methodology for achieving a precise MM diagnosis. The always superior specificity and comparable sensitivity, when compared with dermoscopy alone, suggests the benefit of combined use of these technologies in clinical settings [18,20].

The second study type, prospective interventional, is characterized by the measurement of accuracy in real world settings with clinicians making decisions and bearing legally responsibility while using RCM [6,11,12]. In these studies diagnostic and management decision was taken using all available information including clinical and dermoscopic, explaining the excellent sensitivities and on average a lower specificity. The main outcome measure was reported as the NNE, corresponding to the number of benign lesions excised to detect one MM. Pellacani et al. reported a NNE of 6.8, in contrast to 14.6 without RCM, when RCM was used for management recommendation in a more conservative setting of moderately equivocal lesions [11]. On the other hand, Alarcon et al. stressed the use of RCM in a cohort of dermoscopically positive lesions, reaching a reduction of the NNE from 2.87 to 1.12 [12]. Of note, the recent study by Borsari et al. studied a large sample size of 1279 lesions that underwent RCM examination in a real-world setting because of dermoscopy indication, similar to Alarcon et al., confirming the achievement of an excellent NNE and reported sensitivity of 95.3% [6]. From literature data we can conclude that RCM use in clinical settings is both powerful and cost-effective with the ability to save unnecessary excisions and a very low risk to mismanage a MM, especially evident when applied to dermoscopically positive lesions [27].

However, one limitation for RCM introduction into clinical practice is due to the demonstrated need of dedicated expert training before performing at a clinically comfortable and safe level. Rao et al. reported a good overall diagnostic performance of RCM in a telemedicine setting, but perfect management of all MMs was proposed only by the expert reader whereas the less experienced reader mismanaged 1 out of 9 MMs [14]. A subsequent case revision from the same group showed improvement of diagnostic performance in the same clinical setting after several additional years of RCM practice in a clinical setting [28]. Similarly, Farnetani et al. showed variable accuracy, ranging from a sensitivity of 88.9% and specificity of 79.3%, correlated directly with the expertise of each evaluator [10], confirming the common assertion that RCM is accurate and safe only in expert hands. The need for rapid transfer of essential RCM knowledge and expertise in order to immediately offer the clinical benefits of RCM into practice is supportive of telemedicine application in this field of non-invasive skin cancer screening [29]. However, a telemedicine setting is only able to transfer part of the information and does not permit the "in person patient-doctor experience" which may sometimes be relevant to perceive diagnostic hints and clinical clues able to instinctively direct the clinician to the correct management.

In this study we aimed to test the validity of a RCM telemedicine setting to support diagnosis at a distance and to identify potential strategies that may minimize the risk of MM mismanagement in clinical settings. In our study there were no expert confocal readers involved, but each of the 10 readers received identical dedicated training during a 6 month period for the purpose of future telemedicine evaluation. All 10 readers in our study followed a specific training program that included the individual study and courses entailing dermoscopy and RCM textbooks for skin cancer diagnosis as well as a minimum of 3 months daily exposure to our dermoscopy and RCM outpatient clinic with at least 2000 clinical cases observed and discussed with a tutor.

The dermoscopy-RCM image sets in our study assessed by single reader evaluation reproduced the context of a telemedicine setting and resulted in an overall sensitivity of 95.2% (range: 86 to 100%) and specificity of 76.3% (minimum: 67.5%). Our data is consistent with published literature showing that RCM has a high sensitivity for malignancies: 94.0% for MM, 96.5% for BCC and 87.5% for SCC as well as high specificity for naevi (excluding spitz naevi) (77.5%) and the group of SK/SL/LPLK/AK (79.6%). Overall single reader evaluation resulted in the mismanagement of 8 individual MMs, 4 BCCs and 2 SCCs (based on 2000 total evaluations). These initial results illustrate the potential limitation of single reader evaluation where the diagnostic sensitivity may not be high enough for safe management of MM and allow for potentially dangerous misdiagnosis of lesions that are already invasive or can be life threatening in their progression. This limitation can be attributed to a variety of factors including: lack of sufficient training, limited clinical exposure to RCM, confidence variability, different evaluation criteria followed, fatigue or expediting completion of a high volume of cases in different scenarios.

The addition of a second reader with management for excision made when excision was recommended in at least one of the two evaluations improved the overall sensitivity to 98.3% and lowered specificity to 65.5% (10.8% reduction) resulting in hypothetical mismanagement of only 1 *in-situ* MM and 2 BCCs. The effect of double reader performance decreased the likelihood of mismanagement of an invasive MM in the study population while maintaining an acceptable specificity where the number of unnecessary excision of benign lesions was comparable to that of single reading and to current data from real-world settings [6,11,12]. The mismanaged *in-situ* MM was a diagnostic challenge upon initial presentation. Upon histopathology examination this lesion was characterized by a predominantly lentiginous atypical melanocytic proliferation, consistent with the pattern of lentiginous MM, that represents a slowly progressing variant of MM [30,31]. This lesion was placed into sequential digital dermoscopy follow-up during the first patient visit and was detected during second visit control examination and subsequently removed at that time. The two BCCs cases mismanaged were both small superficial types present on the trunk. Since BCC rarely metastasis these mismanaged false negative lesions would not present significant health risk to these patients and most likely would be diagnosed on a future follow-up digital dermoscopy visit when dermoscopic features would become mature and more apparent.

For individual RCM features we evaluated the frequency of the presence of pagetoid cells, architectural disarray with melanocytic features, BCC tumor islands and/or cords and marked dyskeratosis of the epidermis. Pagetoid cells were reported in 84.3% of MM, 81.2% of spitz naevi and 20.3% of naevi (excluding spitz naevi) as well as architectural disarray with presence of melanocytic features at the DEJ in in 80.1% of MM, 43.8% of spitz naevi and 16.6% of naevi (excluding spitz naevi). These frequencies demonstrate that both pagetoid cells and architectural disarray with melanocytic features at the DEJ are prevalent features of MM. Since both of these features overlap into the category spitz naevi our data is in line with recent publications showing the limitation of RCM in distinguishing spitz naevi from MM, in our study overall single reader specificity was 6.2% and double reader specificity was 0% [32,33]. In the naevi category (excluding spitz naevi) both of these features were present in low but notable frequencies demonstrating their presence in selected groups of melanocytic naevi, particularly those that are congenital, traumatized and/or dysplastic [34,35]. Additionally, the frequencies of BCC tumor islands and/or cords in the BCC population (89.1%) and dyskeratosis in the SCC population (58.3%) were also consistent with literature highlighting their presence as specific for BCC and common in SCC [36–38].

Compared to previously reported methods and technology utilized for skin cancer diagnosis, RCM resulted in a superior combined sensitivity and specificity when applied to dermoscopically equivocal lesions. As examples, multispectral imaging studies reported high sensitivity but have always been associated with poor specificity [39,40]. Monheit et al. performed a prospective, multicenter, blinded study of 1632 lesions and used multispectral imaging to differentiate cutaneous MMs from melanocytic skin lesions using histology as the reference standard. Standard images and patient information for a subset of 50 randomly selected lesions (25 MMs) were used in a reader study of 39 independent dermatologists, resulting in an overall sensitivity of 98.3% and specificity of 9.9% [39]. Lui et al. evaluated 1022 lesions testing Raman-spectroscopy in-vivo and reported that for high sensitivities (95% to 99%) the specificities ranged between 15% and 54% [41], data confirmed in other studies [42,43]. The OCT method is very popular with BCC diagnosis [44,45], but has limited application in differentiating melanocytic tumors due to its lower resolution than RCM [46]. Although Gambichler et al. reported a sensitivity of 74.1% and specificity of 92.4% in the differentiation of cutaneous MM and melanocytic naevi, the authors only included clear MMs and benign lesions, not equivocal ones, which may have given OCT an artificially positive outlook in its application to pigmented lesions [47]. Due to the high resolution and the capability to detect cell metabolic activity, in-vivo multiphoton microscopy is a promising research tool in the field of MM diagnosis [48]. Dimitrow et al. tested in-vivo multiphoton microscopy application in 53 melanocytic lesions (of which there were 26 MMs, 8 of which were in-situ) and reported sensitivity up to 95% and specificity up to 97% in the detection of MM [49]. This technology seems to be very accurate but is limited in clinical settings due to its single point view of a skin lesion prohibiting full-field of view and in time-restrained settings where complete visualization of a cutaneous lesion architecture is necessary to make an accurate diagnosis, mismanagement of MM is possible [50].

In our study it is important to consider the limitation of reader performance based on a retrospective study setting where readers may obtain a higher performance when liability is absent. In consideration of this variable, results in real-world clinical and telemedicine settings may be lower than our own. Therefore, the application of double reading concordance may supplement this potential reduction of performance. Additionally, several malignant cases were managed as benign with high confidence by single reader evaluation when they were in fact malignant and invasive. When the same lesion management was paired in a double reading concordance setting this potentially dangerous mismanagement was reduced due to decision to excise lesions when the double reading was discordant or management decision was concordant for excision.

Double reading enhanced the diagnostic sensitivity and reduced the likelihood to mismanage an invasive MM with the limitation shown to be in *in-situ* lesions, impossible to detect on initial presentation with traditional naked-eye examination as well as a low chance of detection using dermoscopy. These *in-situ* lesions can be well managed with annual screenings and registration of suspicious lesions with sequential digital dermoscopy monitoring. The optimal threshold of safety and accuracy in our study was when two readers managed a skin lesion with concordant benign diagnosis resulting (no-excision) and management of excision when at least one reader was proposing management with excision (discordance). The expected sensitivity/specificity tradeoff must always be considered with any type of screening test and in our study the use of double reading brought sensitivity closer to 100% while reducing specificity from its single reader baseline by only 10.8%. Since an additional benefit of RCM implementation is the reduction of unnecessary excisions, calculated by NNE, this variable should be considered [3].

In conclusion, integration of dermoscopy-RCM imaging is valid in a clinical setting and may be reliable for second expert consultation via telemedicine by adding confidence to equivocal dermoscopic evaluation. The double reader concordance approach may minimize the risk to mismanage a potentially life-threatening skin cancer while maintaining acceptable specificity. Patient screening and sequential digital dermoscopy follow-up should be recommended for all equivocal lesions that are not excised on first visits in order to detect changes over time that may not be visible at initial presentation and initiation of sequential digital dermoscopy (mole-mapping) programs should be considered. Given the high variability of sensitivity and specificity amongst the readers it is important to consider implementation of standardized training and reading programs to help minimize the risk of potential mismanagement using RCM in both clinical and store-and-forward telemedicine settings. Furthermore, a prospective study seems necessary in order to evaluate this promising technology in a real-time telemedicine scenario.

## Supporting information

S1 Table S1_Table.xlsx. (XLSX)
