# Correction: Improving diagnostic sensitivity of combined dermoscopy and reflectance confocal microscopy imaging through double reader concordance evaluation in telemedicine settings: A retrospective study of 1000 equivocal cases

**DOI:** 10.1371/journal.pone.0340306

**Published:** 2026-01-02

**Authors:** A. M. Witkowski, J. Łudzik, F. Arginelli, S. Bassoli, E. Benati, A. Casari, N. De Carvalho, B. De Pace, F. Farnetani, A. Losi, M. Manfredini, C. Reggiani, J. Malvehy, G. Pellacani

The second affiliation for the second author is incorrect. The correct affiliation is not indicated. J. Łudzik is not affiliated with #2 but with: Jagiellonian University Medical College, Department of Bioinformatics and Telemedicine, Kraków, Poland.

The corresponding author’s email address is incorrect. A. M. Witkowsk’s email address is: aw@medvice.health.
